# Malaria and typhoid fever co-infection: a retrospective analysis of University Hospital records in Nigeria

**DOI:** 10.1186/s12936-024-05052-4

**Published:** 2024-07-24

**Authors:** Tubosun A. Olowolafe, Oluwaseyi F. Agosile, Adekemi O. Akinpelu, Nicholas Aderinto, Ojima Z. Wada, David B. Olawade

**Affiliations:** 1 Department of Public Health, Lead City University, Ibadan, Oyo State Nigeria; 2 Department of Epidemiology and Medical Statistics, College of Medicine, University of Ibadan, Ibadan,, Nigeria; 3 Department of Medicine and Surgery, Ladoke Akintola University of Technology, Ogbomoso, Nigeria; 4 Division of Sustainable Development, College of Science and Engineering, Qatar Foundation, Hamad Bin Khalifa University, Doha, Qatar; 5 Global Eco-Oasis Sustainable Initiative (GESI), Ibadan, Oyo State Nigeria; 6 Department of Allied and Public Health, School of Health, Sport and Bioscience, University of East London, London, UK; 7 Department of Research and Innovation, Medway NHS Foundation Trust, Gillingham, ME7 5NY UK

**Keywords:** Malaria, Typhoid fever, Co-infection, Surveillance, Epidemiology

## Abstract

**Background:**

Studies have long documented the presence of malaria and typhoid fever in sub-Saharan Africa (SSA). However, studies on these diseases have primarily concentrated on rural settings, neglecting the potential impact on urban areas. This knowledge gap hinders effective surveillance and intervention strategies. To bridge this gap, this study investigated the prevalence of malaria and typhoid co-infections in an urban environment.

**Methods:**

This study, conducted at Lead City University Hospital in Ibadan, Nigeria (West Africa’s largest metropolis), analysed medical records of over 3195 patients seen between April and June 2023. Descriptive statistics and chi-square tests were used to understand how these co-infections were distributed across different age and gender groups.

**Results:**

The prevalence of co-infection peaked in May (9.7%), followed by June (8.9%) and April (5.7%). Notably, children aged 6–12 years exhibited the highest co-infection rate (18.5%), while those under five had the lowest (6.3%). Gender analysis indicated a slight difference, with 8.8% of females and 7.1% of males co-infected. Malaria prevalence was highest at the beginning of the rainy season and significantly decreased over time. Conversely, typhoid fever displayed the opposite trend, increasing with the rainy season. Children under five years old were most susceptible to malaria, while typhoid fever predominantly affected adults over 25 years old, with prevalence decreasing significantly with age.

**Conclusion:**

This study sheds light on the previously overlooked risk of malaria and typhoid co-infections in urban settings. These findings highlight the need for enhanced surveillance and targeted public health interventions, particularly for vulnerable groups like young children during peak transmission seasons.

## Background

In many developing nations, notably Nigeria, malaria and typhoid fever stand as two predominant infectious diseases that substantially affect public health [1]. Typhoid fever, attributed to the bacterium *Salmonella typhi*, predominantly proliferates through the consumption of tainted food and water. In contrast, malaria results from the *Plasmodium* parasites, transmitted via bites from infected female *Anopheles* mosquitoes. Their endemicity is particularly evident in tropical and subtropical regions due to overlapping geographic distributions [2].

Sub-Saharan Africa bears the brunt of malaria’s prevalence, accounting for an estimated 94% of global malaria cases and fatalities [2]. The ailment poses a grave threat to vulnerable demographics, especially pregnant women and children below five years. In 2019, this paediatric age bracket constituted 67% of global malaria-induced mortalities [2]. Despite persistent mitigation efforts encompassing vector control, prompt diagnostics, and efficacious treatments, malaria persists as a debilitating challenge, causing over 600,000 deaths annually and stymying socioeconomic progression in endemic regions [3].

Concurrently, typhoid fever, afflicting between 11 and 21 million individuals globally each year, results in 128,000–161,000 fatalities [4]. Its prevalence spans vast stretches of Asia, Africa, and Latin America, exacerbated by inadequate sanitation and restricted access to potable water. Although traditionally studied in isolation, evidence suggests that typhoid and malaria can manifest concurrently within a patient, intensifying symptom severity, protracting recovery, and elevating fatality risks [5, 6]. This co-infection poses nuanced challenges for accurate diagnosis, treatment modalities, and comprehensive management [7]. Yet, the interplay and consequent ramifications of this concurrent infection remain under-researched, with most studies adopting a siloed approach, neglecting potential interactions or co-infection outcomes [8].

In communal settings, especially universities, the co-infection of typhoid and malaria stands out as a significant health concern. The close-knit nature of student dormitories and staff quarters can heighten the risk of exposure to these infectious diseases [9]. Such co-infections can drastically impact students’ academic progression and staff productivity. However, it's crucial to recognize that university environments typically comprise a society's more educated echelon. These academic communities, being more informed, often demonstrate better adherence to preventive measures, including rigorous hand hygiene, sanitation practices, and broader health consciousness [10, 11]. Such enhanced awareness and practices might lead to a diminished prevalence of diseases like malaria and typhoid in these populations.

Moreover, urban settings, with their concentrated resources and targeted health interventions, historically exhibit lower disease prevalence [12]. Efforts in these areas often outpace those in rural regions, further widening the rural–urban disparities in Water, Sanitation, and Hygiene (WASH) practices, especially in the studied area [13, 14]. Given these factors, this research delves into the prevalence of malaria, typhoid, and their co-infection in an urban university hospital catering predominantly to academics and their kin. This investigation seeks not only to augment the current understanding of co-infections but also to enrich data repositories on disease prevalence in a socioeconomically advantaged segment of the community. Insights drawn from this study can guide the formulation of targeted preventive strategies, inform policy adjustments, and hone healthcare delivery, ultimately benefiting those grappling with co-infections.

## Methods

### Study design and data source

This retrospective analytical study, utilized patient records from the Lead City University Hospital, Ibadan. The hospital provides a secondary level of care. It is a 30-bed facility with both in-patient and out-patient services. An average number of 70 patients is seen per day by qualified medical personnel. The laboratory section of the facility is headed by a qualified medical laboratory scientist. The laboratory only processes requests from within the hospital. In the facility, malaria and typhoid tests are conducted when requested by physicians. Data were systematically collected from records of patients who visited the hospital laboratory from April to June 2023. The study encompassed a total of 2895 patient records, representing all the patients in the laboratory register during this period. The laboratory records are kept in an Excel sheet. The data extraction process prioritized key variables such as demographic details, clinical symptoms, and diagnostic results.

### Data collection

Relevant data were meticulously extracted from the hospital’s laboratory records using a structured Excel spreadsheet. The extracted variables included age, sex, and diagnostic test results for malaria and typhoid. Age was categorized into brackets (≤ 5, 6–12, 13–19, 20–25, > 25 years), and sex was recorded as male or female. Diagnostic methods comprised blood fluid microscopy examination and serology tests for malaria and typhoid, respectively. Coding in the hospital records identified patients with malaria (coded as 1), typhoid (coded as 2), and co-infection (coded as 3).

### Data analysis

The acquired secondary data were analyzed using IBM SPSS (Statistical Package for the Social Sciences) version 23. The analysis included presenting data through percentages, bar charts, and frequency tables to assess the prevalence and patterns of malaria and typhoid co-infections. In addition, bivariate analysis was conducted via the Chi-square test for independence and was measured at a 95% confidence interval. Statistically significant associations between the diseases surveilled and independent variables like sociodemographic characteristics and infection month were measured following all the test assumptions. This thorough analysis aimed to provide clear insights into the occurrence and distribution of these infections within the studied population.

### Ethical considerations

The study adhered to the Helsinki Declaration principles. Approval was granted by the Health Research Ethics Committee (HREC) of the University and permission was obtained from the Chief Medical Director of the Lead City University Hospital. Throughout the study, stringent measures were taken to ensure compliance with ethical standards and maintain the confidentiality of patient data.

## Results

A total of 2895 patient records were extracted from the laboratory records of those who attended the Lead City Hospital and required laboratory tests done between April and June 2023. There were 32 (1.1%) children under five, 27 (0.9%) patients in the age group 6–12 years, 843 (29.1%) patients in the age group 13–19 years, 757 (26.1%) patients in the age group 20–25 years and 1236 (42.7%) patients above 25 years. There were 1028 (35.5%) patients in April, 1191 (41.1%) in May and 676 (23.4%) in June. Out of these, there were a total of 1078 (37.2%) males and 1817 (62.8%) females (Table 1).

**Table 1:** Background characteristics of patients

Variables	Frequency	Percentage (%)
Age		
Children under five	32	1.1
6–12	27	0.9
13–19	843	29.1
20–25	757	26.1
> 25	1236	42.7
Sex		
Male	1078	37.2
Female	1817	62.8
Month		
April	1028	35.5
May	1191	41.1
June	676	23.4

**Fig. 1:**
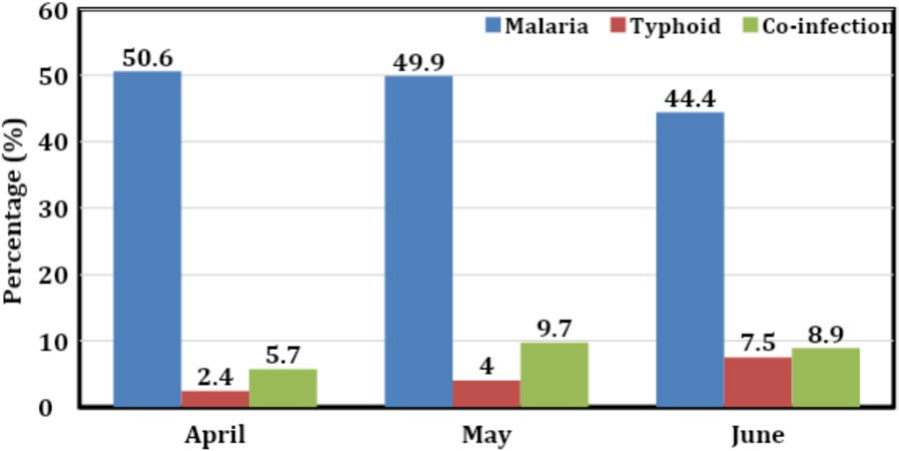
Prevalence of malaria, typhoid and co-infection by months

As revealed in Fig. 1, the prevalence of malaria was 50.6%, 49.9% and 44.4% in April, May, and June, respectively. For typhoid, the prevalence was highest (7.5%) in June, followed by May (4%) and April (2.4%), while the prevalence of co-infection was highest in May (9.7), then in June (8.9%) and (5.7%).

The age group with the highest prevalence of malaria 25 (78.1%) was children under five, while the lowest prevalence of malaria was recorded in the age group 20–25 years with a prevalence of 339 (44.8%). The result showed a significant association (Table 2) between the age groups and the prevalence of malaria (p = 0.002). Females were recorded to have a higher prevalence of 899 (49.5%) compared to males who had a prevalence of 515 (47.8%).

**Table 2:** Prevalence of malaria by background characteristic

	Malaria			
Variable	Positive (%)	Negative (%)	Chi-square	P value
Age			17.184	0.002*
Children under five	25 (78.1%)	7 (21.9%)		
6–12	14 (51.9%)	13 (48.1%)		
13–19	414 (49.1%)	429 (50.9%)		
20–25	339 (44.8%)	418 (55.2%)		
> 25	622 (50.3%)	614 (49.7%)		
Sex			0.786	0.375
Male	515 (47.8%)	563 (52.2%)		
Female	899 (49.5%)	918 (50.5%)		
Month			7.145	0.028*
April	520 (50.6%)	508 (49.4%)		
May	594 (49.9%)	597 (50.1%)		
June	300 (44.4%)	376 (55.6%)		

Table 3 depicts the prevalence of typhoid fever among the patients. A total of 124 patients (4.3%) were positive for typhoid fever. Males had a prevalence of 4.3%, equal to that of females, which was also 4.3%. Although, the relationship between the sexes is not statistically significant (p = 0.974). The highest prevalence, 6.1%, of typhoid fever was observed in patients in the age group > 25, while 6–12 years recorded no typhoid fever infection. The observed difference between them is statistically significant (p = 0.001). The prevalence of typhoid fever was 25 (2.4%) in April, 48 (4.0%) in May and 51 (7.5%) in June. The difference in typhoid fever prevalence was significant (p < 0.001).

**Table 3:** Age and sex prevalence of typhoid fever

Variable	Positive (%)	Negative (%)	Chi-square	P value
Sex			0.001	0.974
Male	46 (4.3%)	1032 (95.7%)		
Female	78 (4.3%)	1739 (95.7%)		
Age			19.762	0.001
Children under five	1 (3.1%)	31 (96.9%)		
6–12	0 (0.0%)	27 (100%)		
13–19	19 (2.3%)	824 (97.7%)		
20–25	29 (3.8%)	728 (96.2%)		
> 25	75 (6.1%)	1161 (93.9%)		
Month			26.316	< 0.001
April	25 (2.4%)	1003 (97.6%)		
May	48 (4.0%)	1143 (96.0%)		
June	51 (7.5%)	625 (92.5%)		

The age prevalence of co-infection with malaria and typhoid fever is shown in Table 4. The age group of 6- 12 years had the highest co-infection of 5 (18.5%), and children under five had the lowest co-infection of 2 (6.3%). However, there was no significant difference in co-infection between the age groups (p = 0.124). Similarly, about 76 (7.1%) of the males and 159 (8.8%) of the females were co-infected with malaria and typhoid fever. However, there was no significant difference (p = 0.105) between the co-infected male and female patients. Table 4 shows that the prevalence of malaria and typhoid co-infection was 59 (5.7%) in April, 116 (9.7%) in May and 60 (8.9%) in June. The difference in prevalence of co-infection in the 3 months was statistically significant (p = 0.002)

**Table 4:** Age and sex prevalence of malaria and typhoid fever co-infection

	Co-infection			
Variable	Positive (%)	Negative (%)	Chi-square	P value
Sex				
Male	76 (7.1%)	1002 (92.9%)	2.623	0.105
Female	159 (8.8%)	1658 (91.2%)		
Age				
Children under five	2 (6.3%)	30 (93.8%)	7.239	0.124
6–12	5 (18.5%)	22 (81.5%)		
13–19	56 (6.6%)	787 (93.4%)		
20–25	66 (8.7%)	691 (91.3%)		
> 25	106 (8.6%)	1130 (91.4%)		

## Discussion

This study utilized data from a private hospital in Ibadan, Nigeria. Given the high endemicity of both malaria and typhoid fever in Nigeria, similar to other tropical and subtropical regions [3, 15, 16], residents are at a significant risk of contracting both diseases simultaneously. The persistence of these diseases as serious public health issues in tropical regions can be attributed to factors such as malnutrition, poor sanitation, inadequate personal hygiene, insufficient healthcare services, and lack of education [17–20].

The findings reveal a high overall prevalence of malaria. This prevalence is higher than that reported in a clinic-based study from Kogi State, which focused on pregnant women attending antenatal clinics [8], but lower than the 60.5% recorded among university students in Akure [9]. The differences in prevalence rates may be influenced by the varying study populations: this study’s hospital-based records likely reflect higher prevalence due to individuals seeking medical care, whereas community-based studies may include asymptomatic or mildly symptomatic individuals, potentially resulting in different prevalence rates.

This study found that females had a higher malaria prevalence, consistent with findings from Sierra Leone, where females also showed a higher prevalence [21]. This could be due to hormonal differences or pregnancy. However, this contrasts with a study from Calabar, which reported a higher prevalence in males [22]. Children under five years had the highest malaria prevalence, followed by the 6–12 age group, aligning with findings from Calabar [22]. This is contrary to the study from FUTA, Akure, which showed the highest prevalence in the 21–25 age group [9]. The high prevalence in young children is likely due to their undeveloped immunity in high transmission areas [23]. Furthermore, malaria prevalence decreased from April (50.6%) to June (44.4%), possibly due to the rainy season’s initial impact on mosquito breeding and subsequent increased prophylaxis measures as the season progressed.

The prevalence of typhoid fever in this study was lower than in studies conducted in Sierra Leone and Calabar, which reported 80.5% and 46.8%, respectively [21, 22]. These comparisons must be contextualized, as this study is based on hospital records, while the Sierra Leone and Calabar studies might include broader community samples. The highest prevalence of typhoid fever was found in patients older than 25 years, which is lower than the 54.5% prevalence in the 31–45 age group reported in Calabar [22]. While this study speculated that the high prevalence in this age group could be linked to contaminated drinking water and poor food hygiene, this study did not collect specific data on these variables, making this assertion speculative. Gender prevalence for typhoid fever was equal in this study, contrasting with findings from Ekwulumili, Anambra State, where females had a significantly higher prevalence [24]. This highlights the need for further research to understand the gender disparities in typhoid prevalence.

Typhoid fever showed an increasing trend from April (2.4%) to June (7.5%), which could be due to the rainy season's effects on water and sanitation conditions, increasing exposure to the pathogen. The overall rate of malaria and typhoid co-infection in this study was higher than that reported in Ekpoma, Edo State [5] but lower than rates found in Aba, Nigeria, and Adamawa, Cameroon, which reported co-infection rates of 40.82% and 30.3%, respectively [25, 26]. The highest co-infection rate was observed in the 6–12 age group, consistent with findings from Cameroon [25]. This could be due to weaker immunity and poor hygiene practices among children, including a lack of understanding of risk factors or increased exposure [10, 27, 28]. Similarly, the age group 1–15 years had the highest co-infection rate in Calabar [22], although their prevalence was higher than in this study. Females had a higher co-infection rate than males, which aligns with studies from Aba and Adamawa [25, 26]. This disparity could be due to socioeconomic factors that predispose females to higher risks. The high malaria prevalence observed in April, May, and June can be attributed to the rainy season, which fosters mosquito breeding due to stagnant water [29].

## Conclusion

This study reveals a distinct pattern of malaria and typhoid fever prevalence within an urban university community in Ibadan, Nigeria, challenging the prevailing assumption that urban areas are less susceptible to these infections. Notably, the prevalence of typhoid fever is relatively low compared to regional averages, possibly due to improved access to clean water and sanitation facilities in the urban university setting. Despite this, the significant rates of malaria and co-infection with typhoid, particularly among children, highlight substantial public health challenges. The heightened vulnerability observed in children is likely linked to their developing immune systems and possibly to socio-economic factors that exacerbate exposure to disease vectors and unsanitary conditions.

The study's findings emphasize the necessity for targeted health interventions that specifically address the needs of vulnerable populations within urban settings. Given the seasonal surge in malaria cases coinciding with the rainy months, proactive strategies to control mosquito breeding, such as environmental management and enhanced community education, should be aggressively implemented. Furthermore, the increasing trend in typhoid fever during the same period underscores the critical need for ongoing improvements in food safety and water quality. Health education campaigns must particularly target at-risk groups, educating them on preventive measures and the critical importance of seeking timely medical attention.

## Data Availability

The datasets used and/or analysed during the current study are available from the corresponding author on reasonable request.

## Abbreviation

WHO: World Health Organization
